# Effectiveness and practicality of control strategies for African swine fever: what do we really know?

**DOI:** 10.1136/vr.103992

**Published:** 2016-11-15

**Authors:** C. Guinat, T. Vergne, C. Jurado-Diaz, J. M. Sánchez-Vizcaíno, L. Dixon, D. U. Pfeiffer

**Affiliations:** 1 Veterinary Epidemiology, Economics and Public Health Group, Royal Veterinary College, Hawkshead Lane, Hatfield, Hertfordshire, AL9 7TA, UK; 2 The Pirbright Institute, Ash Road, Pirbright, Surrey, GU24 0NF, UK; 3 VISAVET Center and Animal Health Department, Veterinary School, Complutense University of Madrid, Madrid, Spain; 4 C. Guinat is also at The Pirbright Institute, Ash Road, Pirbright, Surrey, GU24 0NF, UK

**Keywords:** African swine fever, control strategies, expert elicitation, Best-worst scaling

## Abstract

African swine fever (ASF) is a major pig health problem, and the causative virus is moving closer to Western European regions where pig density is high. Stopping or slowing down the spread of ASF requires mitigation strategies that are both effective and practical. Based on the elicitation of ASF expert opinion, this study identified surveillance and intervention strategies for ASF that are perceived as the most effective by providing the best combination between effectiveness and practicality. Among the 20 surveillance strategies that were identified, passive surveillance of wild boar and syndromic surveillance of pig mortality were considered to be the most effective surveillance strategies for controlling ASF virus spread. Among the 22 intervention strategies that were identified, culling of all infected herds and movement bans for neighbouring herds were regarded as the most effective intervention strategies. Active surveillance and carcase removal in wild boar populations were rated as the most effective surveillance and intervention strategies, but were also considered to be the least practical, suggesting that more research is needed to develop more effective methods for controlling ASF in wild boar populations.

One of the most devastating infectious diseases of pigs is African swine fever (ASF), caused by infection with ASF virus (ASFV). Since its first introduction into Georgia in 2007, ASFV has subsequently spread to neighbouring countries, including Armenia, Azerbaijan, the western parts of the Russian Federation, Iran (2008), Ukraine (2012) and Belarus (2013) (OIE 2007–2013, Rahimi and others 2010). In 2014, the virus entered the EU, with ASF first reported in domestic pigs and wild boar in Lithuania, Poland, Latvia and Estonia (2015) (OIE 2014–2016). Susceptible domestic pigs can become infected by contact with infectious animals, ingestion of contaminated feed, contact with contaminated surfaces and bites from infectious soft ticks (Guinat and others 2016). Trade movements of infectious animals and contaminated pig products, and improper disposal of contaminated carcases also represent important routes for disease spread. There is neither effective treatment nor vaccine available.

According to the Council Directive 2002/60/EC (EC 2002), the suspected presence of ASF in domestic pigs has to be reported immediately to the competent veterinary authority. When the presence of ASFV in a farm is confirmed, 3-km protection and 10-km surveillance zones are implemented around the infected farm. In these zones, pig farms are placed under enhanced surveillance and pig movement restrictions. Intervention strategies include culling of the infected herd and destruction of carcases, followed by outbreak investigations to trace back all movements involving potentially contaminated materials from the infected farm. Repopulation of pig farms cannot take place until 40 days after cleaning and disinfection of the farm. If the presence of ASFV in domestic pigs is confirmed, Member States shall ensure that all pig farms located in the infected area are placed under official surveillance and that a multidisciplinary expert group (involving veterinarians, hunters, wildlife biologists and epidemiologists) assists the competent authority in specifying measures to be implemented (EC 2002). Despite the implementation of these measures, the disease is still prevalent in some EU countries, where between 2014 and 2016, 480 domestic pigs and 3245 wild boar were reported as infected, resulting in 22,360 slaughtered pigs (Empres-i, accessed March 2016).

Recently, expert opinion has been used in several risk assessments on ASFV spread and maintenance in Eastern Europe (EFSA, 2014, 2015, Wieland and others 2011). Their opinions represent useful sources of information when accurate data are lacking (Horst and others 1996, 1998, Garabed and others 2009). A number of methods have been used to elicit expert opinion, including rating (i.e. comparing different items using a common scale) and ranking (i.e. comparing a list of items to one another) scales. However, these classical methods have limitations: they often result in lack of discrimination among items and scale use leads to bias because respondents tend to score items using mostly the scores of the top or bottom of the scale (Jaeger and others 2008, Mielby and others 2012); respondents with the same opinion may score items differently because they interpret the meaning of the scale differently (Austin and others 1998); finally, respondents may have difficulty ranking items when there are a large number of them.

Best-worst scaling (BWS) methodology is an effective approach for measuring the importance of a large number of items (Finn and Louviere 1992). BWS relies on people being generally better at judging items at extremes than in discriminating among items of similar preference (Louviere 1993). In BWS surveys, respondents are asked to identify extremes of their preference (the most and least preferred/important) in repeated and varying sets of a small number of items (generally less than five items to avoid fatigue and confusion of the respondent). Advantages of BWS surveys include questionnaires are relatively easy for respondents to understand. Responses involve choice of items rather than expressing strength of preference, resulting in stronger discrimination among the items and reduced scale use bias. Finally, BWS can be employed even with a large number of items since questionnaires are built with repeated and varying sets of items.

The aim of this study was to review different possible surveillance and intervention strategies for ASF and to assess their effectiveness and practicality as perceived by ASF experts using the BWS methodology.

## Materials and methods

### Selection of experts and shortlisting of control strategies

The group of experts was selected through a snowball sampling method, allowing the number of participants for a survey to increase taking advantage of the social network linkages between experts. An initial group of experts was based on the partners of the EU-funded ASFORCE ‘Targeted research effort on ASF’ project (FP7/2007–2013) (http://asforce.org/). Invited experts were encouraged to suggest additional people with relevant ASF expertise to invite for the survey.

A list of control (surveillance and intervention) strategies for ASF and their definition was generated by reviewing the scientific literature (both published and unpublished). All transmission routes were considered, including via domestic pigs, wild boars, soft ticks, human and environmental components.

Before the survey, the list of control strategies and their definition was reviewed by the experts to ensure that no control strategies of importance for ASF had been omitted and that all definitions were accurate and clear. In total, 35 experts were contacted by email and 14 returned their comments. Tables 1 and 2 describe the 20 surveillance and 22 intervention strategies identified by the expert panel, respectively (see online supplementary appendix for their associated definition).

10.1136/vr.103992.supp1 Supplementary data

**TABLE 1:** The 20 surveillance strategies for African swine fever as identified by the experts’ panel

Item	Surveillance strategy
1	Active surveillance of pigs at abattoirs and rendering plants
2	Active surveillance of pigs at sentinel abattoirs and rendering plants
3	Active surveillance of pigs at farms
4	Active surveillance of pigs at sentinel farms
5	Passive surveillance of pigs at farms
6	Enhanced passive surveillance of pigs at sentinel farms
7	Syndromic surveillance of pig mortality
8	Active surveillance of pig products at butchers, markets and supermarkets
9	Active surveillance of pig products confiscated at the border
10	Active surveillance of fomites
11	Passive surveillance based on inconclusive classical swine fever testing
12	Active surveillance of ticks in tick habitats
13	Active surveillance of ticks in pig farms
14	Active surveillance of ticks in sentinel pig farms
15	Passive surveillance of ticks at farms
16	Enhanced passive surveillance of ticks in sentinel pig farms
17	Active surveillance of wild boar
18	Passive surveillance of hunted wild boar
19	Passive surveillance of wild boar found dead
20	Enhanced passive surveillance of hunted wild boar and wild boar found dead

**TABLE 2:** The 22 intervention strategies for African swine fever listed by the experts’ panel

Item	Intervention strategy
1	Culling of all infected herds
2	Intensive monitoring of neighbouring herds
3	Culling of neighbouring herds
4	Intensive monitoring of traced herds
5	Culling of traced herds
6	Culling of neighbouring or traced herds followed by heat treatment and consumption
7	Movement bans for neighbouring herds
8	Movement bans for traced herds
9	Ban of swill feeding
10	Thorough cleaning and disinfection of buildings, transport vehicles and personal protective equipment
11	Health and safety regulations on farms
12	Farm entrance restrictions on people
13	Containment of pigs
14	Ban of live animal markets
15	Health and safety regulations at border
16	Ban of large-scale drive hunting of wild boar
17	Supplementary feeding of wild boar
18	Ban of supplementary feeding of wild boar
19	Targeted hunting of wild boar
20	Carcase removal of wild boar
21	Exclusion/fencing of wild boar
22	Wild boar deterrents

### Questionnaire design

The questionnaire was designed using the Survey Gizmo software (www.surveygizmo.eu/) and was accessible online. The purpose of the study, use of the data, respondents’ anonymity and expected completion time (15 minutes) were explained at the beginning of the survey. A pilot study was conducted with four scientists from the ASFORCE project to ensure the objective of the study and the questions were clear and easy to understand and to determine the time needed to complete the survey. Experts were invited by email to participate in the study (August 28, 2015), with a reminder email sent one week before the survey completion date (September 29, 2015).

The survey had four sections: effectiveness of surveillance strategies, practicality of surveillance strategies, effectiveness of intervention strategies and practicality of intervention strategies. A ‘practical’ strategy was defined as being feasible under real-world circumstances, taking into account considerations such as cost, logistics and acceptability. For example, strategies may be easy to implement in theory (e.g. ban of on-swill feeding) but in reality people may not be willing to comply, and the strategy therefore would not be considered practical. An ‘effective’ strategy was defined as expected to be successful in producing the intended objectives, which were to detect ASF outbreaks as early as possible (for surveillance strategies) or to reduce the likelihood of ASFV introduction and spread (for intervention strategies). Experts were asked to assess the strategies within a ‘European context’, that is, assuming a relatively good veterinary infrastructure, a high level of public trust towards authorities and availability of sufficient economic resources.

### BWS methodology

BWS methodology was used to elicit expert opinion regarding the effectiveness and practicality of the different measures for controlling ASF. The experts were presented with sets consisting of four different surveillance and intervention strategies. For each set, respondents were asked to indicate ‘the most effective’ and ‘the least effective’ strategy (as well as ‘the most practical’ and ‘the least practical’ strategy) among the four strategies. A choice of four strategies for each set was considered to provide a good compromise between variety of options and time required to choose. The importance of separately considering ‘effectiveness’ and ‘practicality’ for each set was emphasised with each question as it was expected that respondents otherwise would tend to choose the best and worst strategies based on an unintentional integration of effectiveness and practicality.

### Data analysis

Only responses from participants who completed all questions were included in the analysis. The data were analysed using a scaled simple count method (Finn and Louviere 1992, Auger and others 2007) available in the Survey Gizmo software. This allowed for each participant to generate a score for each surveillance and intervention strategy based on the number of times it was considered as ‘the most effective/practical’ and ‘the least effective/practical’ strategy. The overall relative importance of each strategy was calculated as the average of its scores across all respondents. To facilitate interpretation of the results, average scores were centred and normalised and the strategies were plotted in a practicality and effectiveness space x-y chart where the axes represent the average effectiveness and practicality scores across respondents. Strategies plotted above zero on the y-axis showed a higher effectiveness score than the average, and those plotted above zero on the x-axis indicated a higher practicality score than the average. For each strategy that was scored above average both for effectiveness and practicality, an integrated measure of the effectiveness and the practicality was calculated as the distance between the strategy's coordinates and the axes origin. Level of agreement between respondents was assessed using Kendall's W, which is a non-parametric measure of concordance between raters. Values of W under 0.26, between 0.26 and 0.38, and over 0.38 were interpreted as weak, moderate and strong agreement, respectively (Siegel and Castellan 1988).

## Results/discussion

The final survey was sent to 56 experts. Out of 56, 35 were members of the ASFORCE project. Fifty experts participated in the survey giving a response rate of 89.0 per cent. Twenty-nine completed all questions and were therefore included in the analysis. These respondents originated from Italy (6/29), United Kingdom (4/29), South Africa (2/29), Belgium (2/29), France (2/29), Germany (2/29), Spain (2/29), Bulgaria (1/29), Ethiopia (1/29), Madagascar (1/29), Mexico (1/29), the Netherlands (1/29), the Russian Federation (1/29), Sweden (1/29), Switzerland (1/29), and United States of America (1/29). Nine (31.0%) of them were from countries where ASF outbreaks occurred over the last 12 months (WAHIS Interface, accessed Aug 2016).

**FIG 1:**
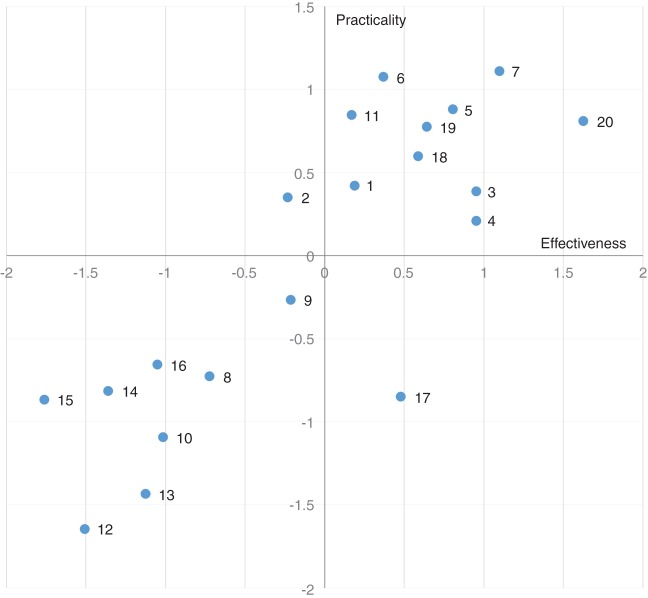
Zero-centred scatterplot of effectiveness and practicality scores for the 20 surveillance strategies for African swine fever. The level of agreement among respondents was strong (W=0.382) and moderate (W=0.342) with respect to the effectiveness and practicality of the surveillance strategies, respectively. The plot shows that 10 strategies are scored above average for both effectiveness and practicality (those located in the upper-right quadrant) and are therefore considered to be optimal for surveillance (Table 3)

Enhanced passive surveillance aimed at hunted wild boar and wild boar carcases (no. 20) and syndromic surveillance of pig mortality in farms (no. 7) were regarded as the optimal surveillance strategies for detecting ASFV (Fig 1). Accordingly, 96.9 per cent (31 out of 32) of infected pig farms in Latvia were detected by passive surveillance compared with 2.1 per cent having been detected by active surveillance (Oļševskis and others 2016). Disease surveillance in wild boar was regarded as a very important strategy since they were the first ASF cases reported in the affected EU countries (Estonia, Latvia, Lithuania and Poland) (Gavier-Widén and others 2015). Similarly, 71.4 per cent (175 out of 245) of wild boar carcases were reported positive for ASFV infection in Latvia compared with 1.5 per cent (41 out of 2,765) of hunted wild boar (Oļševskis and others 2016). This could be explained by the fact that infected domestic pigs and wild boar have mainly developed the acute form of the disease, leading to sudden death within 5–13 days, before the detection of any suspect clinical signs (Gabriel and others 2011, Blome and others 2012, Guinat and others 2014, Gallardo and others 2015, Pietschmann and others 2015). Also, recent diagnostic investigations in Latvia and Poland have indicated that the majority of infected domestic pigs and wild boar died before the detection of any seropositive animals, that is, within 7–10 days after infection (Oļševskis and others 2016, Woźniakowski and others 2016). These results suggest that disease surveillance and detection primarily relies on accurate and timely mortality reports from pig farmers and hunters before the disease can be diagnosed on the basis of the clinical signs or the use of immunological tests. However, it has been pointed out that a strategy based on passive surveillance would only be effective if farmers’ and hunters’ disease awareness is sufficiently high that a suspicion based on observed mortality is followed by timely reporting to the competent authorities. For example, a study conducted in Bulgaria, Germany and the Russian Federation showed that most of the farmer and hunter respondents were willing to comply with reporting requirements for ASF (although this does, of course, not guarantee their actual compliance in the event of an outbreak) (Vergne and others 2014). Also, 87 per cent (211 out of 243) of farmers would immediately report an ASF suspicion, 52 per cent (189 out of 366) of hunters would subject hunted wild boar to diagnostic testing and 83 per cent (123 out of 149) of hunters would report wild boar found dead (Vergne and others 2014). Active surveillance of pigs at farms (no. 3) was also suggested as an optimal strategy (Table 3) although ranked at lower values than passive surveillance as it could potentially help in the detection of positive farms in areas at high risk of infection. That is probably because both field observations and experimental studies have shown that ASFV transmission from pig to pig is low during the early stage of the infection, resulting in the death of only a small number of pigs during this time period (Guinat and others 2016, Oļševskis and others 2016), which will complicate disease surveillance and detection within a herd. In addition, serological results from field studies in Russia, Latvia and Poland have shown that some animals could remain infected for longer than two weeks (Mur and others 2014, Oļševskis and others 2016, Woźniakowski and others 2016). One experimentally infected pig was reported asymptomatic with intermittent viraemia for up to 38 days post exposure using Lithuania ASFV strain (Gallardo and others 2015). Thus, long-term asymptomatic carriers may influence future disease surveillance guidelines.

**TABLE 3:** Optimal surveillance and intervention strategies for African swine fever

Item	Optimal strategy	Rank (distance)
*Surveillance*		
20	Enhanced passive surveillance of hunted wild boar and wild boar found dead	1 (1.82)
7	Syndromic surveillance of pig mortality	2 (1.56)
5	Passive surveillance of pigs at farms	3 (1.20)
6	Enhanced passive surveillance of pigs at sentinel farms	4 (1.14)
3	Active surveillance of pigs at farms	5 (1.03)
19	Passive surveillance of wild boar found dead	6 (1.01)
4	Active surveillance of pigs at sentinel farms	7 (0.98)
11	Passive surveillance based on inconclusive classical swine fever testing	8 (0.86)
18	Passive surveillance of hunted wild boar	9 (0.84)
1	Active surveillance of pigs at abattoirs and rendering plants	10 (0.46)
*Intervention*		
1	Culling of all infected herds	1 (9.59)
7	Movement bans for neighbouring herds	2 (5.99)
13	Containment of pigs	3 (5.83)
11	Health and safety regulations on farms	4 (5.73)
2	Intensive monitoring of neighbouring herds	5 (5.45)
8	Movement bans for traced herds	6 (5.13)
12	Farm entrance restrictions on people	7 (4.78)
4	Intensive monitoring of traced herds	8 (3.24)
10	Thorough cleaning and disinfection of buildings, transport vehicles and personal protective equipment	9 (1.99)
9	Ban of swill feeding	10 (1.77)

Surveillance strategies aimed at ticks (no. 12–15), pig products (nos. 8 and 9) and fomites (no. 10) were regarded as the least effective and practical (Fig 1). More research is required to understand their potential epidemiological roles (i.e. tick presence, infectious dose for contaminated feed ingestion and fomites, etc) before they can be considered for integration in surveillance strategies. The strategy located in the lower-right quadrant (Fig 1), that is, active surveillance of wild boar (no. 17), was perceived as fairly effective but of poor practicality. However, the use of non-invasive sampling (such as oral fluid and faeces collection) has been suggested since it is less costly, less dangerous and less logistically feasible compared with trapping and hunting wild boar (de Carvalho Ferreira and others 2014, Petrov and others 2014). The rope-based sampling technique has also been developed for oral fluid collection and for early detection of swine diseases in wild boar (Mouchantat and others 2014a, b).

Consistent with European legislation (Council Directive 2002/60/EC), in this study culling of all infected herds (no. 1), movement bans (nos. 7 and 8) and intense monitoring (nos. 2 and 4) for neighbouring and traced herds were perceived as optimal intervention strategies (Table 3) for reducing the likelihood of ASFV introduction and spread. Containment of pigs (no. 13), ban of swill feeding (no. 9) and entrance restrictions for farm visitors (no. 12) were also viewed as optimal intervention strategies (Table 3), probably because they represent some of the major causes of outbreaks in pig farms. For example, 42.9 per cent (12 out of 28) and 57.1 per cent (16 out of 28) of primary outbreaks in Latvia were related to contact with wild boar and swill feeding practices, respectively, while 100.0 per cent (4 out of 4) of secondary outbreaks were related to entrance of visitors with previous contacts of infected farms (Oļševskis and others 2016). Biosecurity measures on farms and at the farm entrance (such as thorough cleaning and disinfection of buildings, transport vehicles and personal protective equipment (no. 10), and health and safety regulations on farms (no. 11)) were seen as optimal (Table 3), although their role in the introduction of ASFV remains difficult to quantify.

**FIG 2:**
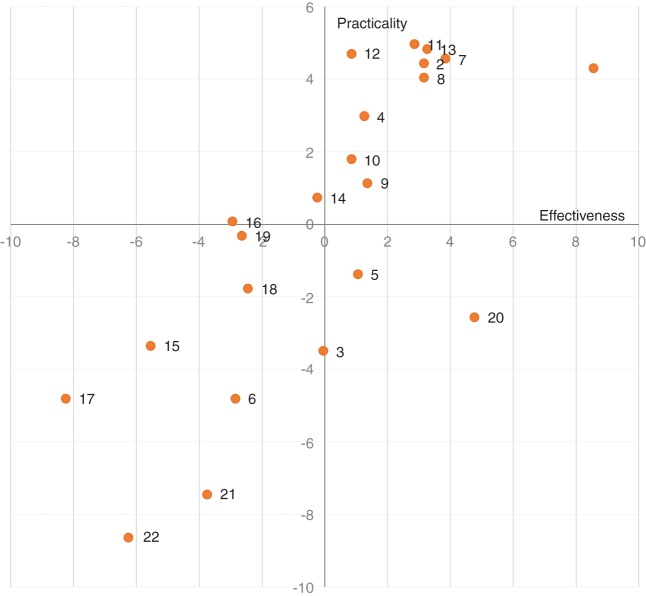
Zero-centred scatterplot of effectiveness and practicality scores for the 22 intervention strategies for African swine fever. The level of agreement among respondents was weak with respect to both the effectiveness (W=0.216) and practicality (W=0.136) of the intervention strategies. The figure shows that 10 strategies are scored above average both for effectiveness and practicality (those located in the upper-right quadrant) and are therefore considered to be optimal for intervention (Table 3)

Most of the interventions that could be implemented on wild boar were regarded as less effective and practical than the average due to the challenges of monitoring wild boar populations. Both use and ban of supplementary feeding for wild boar (nos. 17 and 18) were scored as less effective and practical than the average (Fig 2), probably because these interventions remain controversial. While the practice of supplementary feeding may increase the population contact rate and facilitate ASFV spread (Gavier-Widén and others 2015), others have argued that supplementary feeding may perform well in some circumstances (depending on seasons and sites) for ASF control. It was suggested that feeding could increase the population contact rate and wild boar would become infected and die in the local area rather than spread the disease to other populations (Geisser and Reyer 2004). Both strategies related to hunting of wild boar (nos. 16 and 19) were also viewed as less effective and practical than the average (Fig 2). Research is still needed to understand the effect of targeted hunting on wild boar density (Gavier-Widén and others 2015). A ban of large-scale hunting of wild boar (no. 16) was not considered to be an optimal strategy. This hunting practice is believed to be one of the main drivers of ASFV spread from Belarus to Poland and Lithuania and from the Russian Federation to Ukraine. Strategies located in the lower-right quadrant (Fig 2), such as removal of wild boar carcases (no. 20), were perceived as relatively effective but of little practicality. However, it has been suggested that the use of trained dogs for searching of carcases could increase the practicality of this strategy (Selva and others 2005) and that payment for wild boar carcases could motivate hunters to contribute to ASF control (Gavier-Widén and others 2015). The intervention strategy involving culling of pig herds followed by heat treatment of meat (no. 6) and its use in the human or pet food chain was presented to experts as an alternative strategy. This could be a way for farmers to reduce the economic impact generated by culling of pig herds, particularly in countries where no funds for financial compensation are available (Thomson and others 2013). However, this strategy was perceived as being poorly effective and practical (Fig 2), probably due to potential food safety risks. Fences around areas for pig keeping to prevent wild boar access (no. 21) were neither seen as effective nor as practical (Fig 2). A proposal to build a fence along the border of Lithuania with Belarus had not been supported by the EC (PROMED 2014). Health and safety regulations at the border (no. 15) were also considered to be poorly effective and practical (Fig 2). Since the first cases reported in Belarus in 2013, affected EU countries have carried out cleaning and disinfection of livestock transport vehicles at the borders with Belarus and the Russian Federation (EC 2013), although these procedures were reduced during winter months due to adverse weather conditions. Controls have also been conducted on personal luggage for products of animal origin, which has led to the seizure of nearly 20 tonnes of such material in 2013. Limited data are available on the number of samples from meat products detected positive for ASFV that were collected as part of this surveillance. Until now, Latvian veterinary authorities reported 6 out of 42 samples of meat products positive for ASFV genome (EC 2015).

The results presented here reflect the perception of the experts who were interviewed. Therefore, they will be influenced by variation in knowledge (laboratory vs epidemiology) among experts and previous experience with ASF. For example, we expect that experts originating from countries where ASF outbreaks are occurring or who experienced ASF in the field have a more critical opinion on the disease control compared with those who had never been directly involved in ASF control and therefore may be more likely to support the official guidelines. In addition, the survey did not account for the local context (such as an area with both wild boar and domestic pigs present versus an area with only domestic pigs or only wild boar) and farm types (such as large commercial farm, traditional free-ranging and backyard pig farms). Thus, these results provide broad guidance in relation to optimal strategies in a general ‘European’ context, recognising that there is likely to be variation between different epidemiological country-specific scenarios. It is likely that for this reason participants who did not complete all survey questions commented that they found the task too difficult.
