# Supplementary material for: Effectiveness and practicality of control strategies for African swine fever: what do we really know?
Source: Vet Rec. 2016 Nov 15;180(4):97. doi: 10.1136/vr.103992 (PMC5293861; doi:10.1136/vr.103992)
Supplement: Supplementary data [file vetrec-2016-103992supp.pdf]

Table 1. The 20 surveillance strategies for ASF as identified by the experts' panel

Item	Surveillance strategy	Description
1	Active surveillance of pigs at abattoirs and rendering plants	This consists in the repeated collection of data from randomly selected abattoirs and rendering plants to identify changes in pig health status (i.e. performing clinical inspections, sample collection, laboratory tests, etc.) The difference with "*active surveillance of pigs at sentinel abattoirs and rendering plants* " is that here, the abattoirs and rendering plants are expected to change over time
2	Active surveillance of pigs at sentinel abattoirs and rendering plants	This consists in the repeated collection of data from selected abattoirs and rendering plants to identify changes in pig health status in abattoirs and rendering plants (i.e. performing clinical inspections, sample collection, laboratory tests, etc.) The difference with "*active surveillance of pigs at abattoirs and rendering plants*" is that here, the selected abattoirs and rendering plants are expected to remain the same over time and have been identified as high risk of disease introduction
3	Active surveillance of pigs at farms	This consists in the repeated collection of data from randomly selected farms to identify changes in pig health status (i.e. performing clinical inspections, sample collection, laboratory tests, etc.) The difference with "*active surveillance of pigs at sentinel farms*" is that here, the farms are expected to change over time
4	Active surveillance of pigs at sentinel farms	This consists in the repeated collection of data from selected farms to identify changes in pig health status in farms (i.e. performing clinical inspections, sample collection, laboratory tests, etc.) The difference with "*active surveillance of pigs at farms*" is that here, the selected farms are expected to remain the same over time and have been identified as high risk of disease introduction
5	Passive surveillance of pigs at farms	Farmers and animal workers report voluntarily the suspicion of ASF on their farm to the competent authority. Suspicion can come from the close monitoring of pig production data (e.g. syndromic surveillance mortality at farm level).
6	Enhanced passive surveillance of pigs at sentinel farms	Farmers and animal workers report voluntarily the suspicion of ASF on their farm to the competent authority The difference with "*passive surveillance of pigs at farms*" is that here, farms identified as high risk of disease introduction, are more likely to report a suspicion. Enhancement may come from financial incentives, improved awareness of ASF clinical signs, current legislation, etc.

7	Syndromic surveillance of pig mortality	Systematic collection and monitoring of pig mortality data at farm level to detect any unusual increase.
8	Active surveillance of pig products at butchers, markets and supermarkets	This consists in the repeated collection of data from pig products at butchers, markets and supermarkets to identify changes in pig product status (i.e. performing sample collection, laboratory tests, etc.) and to detect if infected pig products have entered the food chain
9	Active surveillance of pig products confiscated at the border	This consists in the repeated collection of data from pig products at airports, ports and land borders, particularly when originating in infected countries.
10	Active surveillance of fomites	This consists in the repeated collection of data from trucks that transport pigs, but also agricultural products that could be infected (feedstuff) to identify virus contamination
11	Passive surveillance based on inconclusive CSF testing	Investigation of classical swine fever-suspect farms for which a confirmation or laboratory diagnosis has not been reached
12	Active surveillance of ticks in tick habitats	This consists in the repeated collection of ticks in tick habitats (such as rodent burrows etc.) for being tested for ASF diagnosis
13	Active surveillance of ticks in pig farms	This consists in the repeated collection of ticks in <u>randomly selected</u> pig farms for being tested for ASF diagnosis.
14	Active surveillance of ticks in <u>sentinel</u> pig farms	This consists in the repeated collection of ticks in <u>selected</u> pig farms for being tested for ASF diagnosis<br>The difference with "*active surveillance of ticks at farms*" is that here, the selected farms have been <u>identified as high risk</u> for ASFV maintenance in ticks because of contacts between ticks and pigs have been identified by performing serological testing against tick bite in domestic pigs (to find antibodies against the salivary glands of *Ornithodoros* ticks)
15	Passive surveillance of ticks at farms	Farmers and animal workers report voluntarily the presence of ticks on farm to the competent authority for being tested for ASF diagnosis
16	<u>Enhanced</u> passive surveillance of ticks in <u>sentinel</u> pig farms	Farmers report voluntarily the presence of ticks on <u>selected</u> farm to the competent authority for being tested for ASF diagnosis<br>The difference with "*passive surveillance of ticks at farms*" is that here, farms <u>identified as high risk</u> for ASFV maintenance in ticks, are more likely to report the presence of ticks. These farms were <u>selected</u> because of contacts between ticks and pigs have been identified by performing serological testing against tick bite in domestic pigs (to find antibodies against the salivary glands of *Ornithodoros* ticks). <u>Enhancement</u> may come from financial incentives, improved awareness of ASF clinical signs, current legislation, etc.
17	Active surveillance of wild boar	This consists in the repeated captures (by trapping, hunting, etc.) of wild boar to identify changes in wild boar health

		status (i.e. performing clinical inspections, sample collection particularly with the use of non-invasive sampling, laboratory tests, etc.)
18	Passive surveillance of hunted wild boar	Hunters report voluntarily hunted wild boar to the competent authority for collection of samples being tested for ASF diagnosis
19	Passive surveillance of wild boar found dead	Hunters (but also farmers, walkers, etc.) report voluntarily wild boar found dead to the competent authority for collection of sample being tested for ASF diagnosis
20	Enhanced passive surveillance of hunted wild boar and wild boar found dead	Hunters (but also farmers, walkers, etc.) of selected areas report voluntarily hunted wild boar and wild boar found dead to the competent authority for being tested for ASF diagnosis<br>The difference with "*passive surveillance of wild boar*" is that here, selected forests have been identified as high risk for disease introduction. Enhancement may come from financial incentives, improved awareness of ASF clinical signs, current legislation, etc.

Table 2. The 22 intervention strategies for ASF listed by the experts' panel

Item	Intervention strategy	Description
1	Culling of all infected herds	All infected herds are culled. This also includes proper disposal of all dead pigs and financial compensation for culled and dead pigs
2	Intensive monitoring of neighbouring herds	Herds, which are located within a defined radius around infected herds, are subjected to intensive monitoring of mortality. This is an alternative to "*culling of neighbouring herds*"
3	Culling of neighbouring herds	Culling of herds which are located within a defined radius around infected herds. This also includes proper disposal of all culled pigs. This is an alternative to "*intensive monitoring of neighbouring herds*"
4	Intensive monitoring of traced herds	Herds, which did trade pigs with the infected herds, are subjected to intensive monitoring of mortality. This is an alternative to "*culling of traced herds*"
5	Culling of traced herds	Culling of herds which did trade pigs with the infected herds. This also includes proper disposal of all culled pigs. This is an alternative to "*intensive monitoring of traced herds*"
6	Culling of neighbouring or traced herds followed by heat treatment and consumption	This is an alternative strategy in countries where there are no funds for financial compensation and so where reporting of outbreaks by the farmers are completely discouraged. Heat processing of the culled animals into sausages, canned meat, etc. would allow some sort of compensation.
7	Movement bans for neighbouring herds	Ban on animal movements (and products) for herds which are located within a defined radius around

		infected herds. This might be associated with intensive monitoring of neighbouring herds.
8	Movement bans for traced herds	Ban on animal movements (and products) for herds which did trade pigs with the infected herds
9	Ban of swill feeding	Pigs should be not fed with swill that might contain contaminated remains of pigs. This also includes proper disposal of waste food
10	Thorough cleaning and disinfection of buildings, transport vehicles and PPE	From farm-to-farm and from abattoir-to-farm. PPE: Personal Protective Equipment, e.g. clothing, boots, masks, etc.
11	Health and safety regulations on farms	This consists in improving the sanitary barriers: quarantine of pigs at farm entrance (i.e. physical isolation of pigs entering the farm for period of time) and measures to ensure the safe purchase of pigs, (such as vet inspection certificates, testing of pigs, adoption of basis measures of hygiene, etc.). This also implies improved awareness of farmers on ASF clinical signs.
12	Farm entrance restrictions on people	Restrictions for public access in all farms. Access to farmland (via footpaths) restricted during outbreaks
13	Containment of pigs	Installation of pens and barriers in all farms for the prevention of contacts with wild boar and reduction of scavenging behaviour. This includes the ban of free ranging pig
14	Ban of live animal markets	This could be either a permanent measure or a temporary measure when outbreaks are reported in the area
15	Health and safety regulations at border	This is to prevent the entry of the disease into a zone with luggage inspection, use of dogs, awareness of passengers through posters, random inspections, etc.
16	Ban of large-scale drive hunting of wild boar	This is a ban on the massive depopulation of wild boar
17	Supplementary feeding of wild boar	This consists in supplying feed to wild boar to attract them for contact hunting purposes<br>This could be used to increase the contact rate and disease transmission in sub-populations. This sub-population would quickly die in a restricted area rather than spreading the disease to nearby populations.
18	Ban of supplementary feeding of wild boar	This is a ban on the provision of limited supply of feed to attract wild boar for contact hunting purposes
19	Targeted hunting of wild boar	The size of the hunting bag remains similar although the population of piglets at early ages and adult females is targeted for hunting in order to reduce the population reproduction rate. This also consists in reducing the density population before the risk of disease introduction increases in order to reduce the likelihood of disease spread.
20	Carcass removal of wild boar	This means the collection of carcasses and proper disposal of them

21	Exclusion/Fencing of wild boar	This consists in the installation and maintenance of (electric) fences to prevent wild boar from entering an area
22	Wild boar deterrents	This consists in the installation of devices (ex. scare crows) and repellents (ex. olfactory, gustatory) to make wild boar move away from farms
